# Biocomposites Based on Poly(3-Hydroxybutyrate-*co*-3-Hydroxyvalerate) (PHBHV) and *Miscanthus* *giganteus* Fibers with Improved Fiber/Matrix Interface

**DOI:** 10.3390/polym10050509

**Published:** 2018-05-07

**Authors:** Erica Gea Rodi, Valérie Langlois, Estelle Renard, Vittorio Sansalone, Thibault Lemaire

**Affiliations:** 1 Laboratoire de Modélisation et Simulation Multi-Echelle, Université Paris Est, UMR 8208, CNRS 61 Avenue du Général de Gaulle, 94010 Crétéil, France; rodi@icmpe.cnrs.fr (E.G.R.); vittorio.sansalone@u-pec.fr (V.S.); 2 Institut de Chimie et des Matériaux Paris-Est, Université Paris Est, UMR 7182, CNRS, 2-8 rue Henri Dunant, 94320 Thiais, France; renard@icmpe.cnrs.fr

**Keywords:** biocomposites, reactive extrusion, interface improvement, mechanical characterization, multiphase models

## Abstract

In this paper, green biocomposites based on poly(3-hydroxybutyrate-*co*-3-hydroxyvalerate) (PHBHV) and *Miscanthus giganteus* fibers (MIS) were prepared in the presence of dicumyl peroxide (DCP) via reactive extrusion. The objective of this study was to optimize the interfacial adhesion between the reinforcement and the matrix, improving the mechanical properties of the final material. To this aim, two fibers mass fractions (5 and 20 wt %) and two different fiber sizes obtained by two opening mesh sieves (1 mm and 45 μm) were investigated. The impregnation of fibers with DCP before processing was carried out in order to promote the PHBHV grafting onto MIS fibers during the process, favoring, in this way, the interfacial adhesion between fibers and matrix, instead of the crosslinking of the matrix. All composites were realized by extrusion and injection molding processing and then characterized by tensile tests, FTIR-ATR, SEM, DSC and XRD. According to the improved adhesion of fibers to matrix due to DCP, we carried out an implementation of models involving that can predict the effective mechanical properties of the biocomposites. Three phases were taken into account here: fibers, gel (crosslinked matrix), and matrix fractions. Due to the complexity of the system (matrix–crosslinked matrix–fibers) and to the lack of knowledge about all the phenomena occurring during the reactive extrusion, a mathematical approach was considered in order to obtain information about the modulus of the crosslinked matrix and its fraction in the composites. This study aims to estimate these last values, and to clarify the effect caused by the presence of vegetal fibers in a composite in which different reactions are promoted by DCP.

## 1. Introduction

In recent years, the attention of both academia and industry was focused on eco-friendly materials from renewable resources, due to the growing concern over environmental issues. The pollution caused by non-biodegradable synthetic plastics led to the investigation of totally biobased polymers that could replace the first ones [1,2,3,4,5,6,7]. A class of polyesters of great interest is microbial polymers known as poly(3-hydroxyalkanoate)s (PHAs). This family of polymers is known for the good biodegradability and biocompatibility, being derived from bacterial synthesis [8,9,10]. These polymers have been used for a wide range of applications, starting from biomedical, such as for tissue engineering and bone replacement, to packaging, agriculture, and personal disposal articles [8,11,12,13].

Among PHAs, two of the major polymers that have been largely investigated are poly(3-hydroxybutyrate) (PHB) and poly(3-hydroxybutyrate-*co*-3-hydroxyvalerate) (PHBHV). The difficulty in processing and the very brittle character of PHB are two reasons explaining our interest on the second polymer. Notwithstanding its better flexibility due to the HV units, its high cost limits the usage of PHBHV in industrial applications. In this context, green composites associating such biobased polymers and vegetal fibers constitute an attractive alternative, due to their lower cost, biodegradability, renewability, and pretty good mechanical properties [14,15,16,17,18,19,20,21,22,23,24,25,26,27]. 

Among all vegetal fibers, *Miscanthus giganteus* presents many advantages, like the possibility to be cultivated on poor quality soil and in the presence of very little amount of herbicide and water [28,29,30], the high productivity in particular in temperate climates [31,32], with good yield also in relatively cold zones [33]. These features and the variegated mechanical properties [34,35] make *Miscanthus* a good candidate for the production of fuels and chemicals [36], and also, as reinforcement [37]. Although, all these advantages, the hydrophilic character of vegetal fibers, and the hydrophobic one of biobased polyesters, affect the transmission of the stress, resulting in poor mechanical properties [38].

Interfacial adhesion could be improved by different techniques that have already been tested [39,40,41,42,43,44,45,46,47]. Recently, the free radical grafting initiated by peroxides has been largely used in different polymeric blends to induce coupling between molecular chains [44,48], but also to graft cellulosic fibers or wood derivatives to different matrixes [49,50,51,52,53]. Although the biocomposites realized in precedent works were fully characterized by mechanical and thermal tests, indicating a decrease in crystallinity after addition of the reinforcement and DCP, no studies were carried out to predict the effective mechanical behavior of these types of composites with compatibilized interface matrix/fibers. 

Numerical models based on a kinetic approach were implemented in past years, to predict the molecular weight distribution, as a function of time, for polyolefin crosslinked in the presence of peroxides [54,55,56,57]. Other models based on statistical approaches [58,59,60] and Monte Carlo simulations tried to predict a series of reactions that may occur during the crosslinking phenomenon, in order to fully characterize this complex process [61]. Among all these methods, kinetics models were preferred to statistical ones, due to the impossibility of the latter to predict the reactions as a function of time, and only as function of conversion. However, in such cases, the mathematical analyses implemented were much too simplistic for real polymers, or much too specific to particular compositions or reactions [61]. In this study, we proposed an approach based on the reactive grafting initiated by DCP, in order to improve the adhesion of *Miscanthus* fibers to a PHBHV matrix, with a consequent improvement of the mechanical properties of the final composite. The reaction was conducted in situ using extrusion followed by injection mold processing, and the final materials were fully characterized to detect any possible change in the crystalline structure. Thus, it was shown that DCP induced a crosslinking process within the matrix such that, in addition to the matrix phase, a gel (crosslinked matrix) with different mechanical properties should be taken into account to understand the overall mechanical behavior of the composite.

Starting from the experimental procedure, modeling strategies based on mixing rule and the inclusion theory of Eshelby were thus proposed to calculate the effective elastic behavior of a composite realized by this technique. The confrontation between experimental and model results does not provide a perfect agreement, showing that the complexity of the system and the lack of knowledge about crosslinking and grafting phenomena occurring during the reactive process needs to be clarified more, in detail. In particular, the elastic properties of the crosslinked matrix and its exact fraction in the composites constitute two great limits for the implementation of realistic models. To this aim, a mathematical approach was considered in order to evaluate the change of the elastic modulus of the crosslinked matrix as function of its fraction in the composites. This procedure clarifies the role of DCP and that of fibers in the reactive blend, quantifying a range of crosslinked fraction matrix generated by a given content of DCP and for a given content of fibers.

## 2. Materials and Methods

### 2.1. Materials

Poly(3-hydroxybutyrate-*co*-3-hydroxyvalerate) (PHBHV) containing 12% of valerate was purchased from Goodfellow, Lille, France. *Miscanthus giganteus* (MIS) was provided by Miscanplus, France. It came from a 2014 spring crop roughly chopped and subsequently milled with two different sieves with an opening mesh of 1 mm and 45 µm. For simplicity, in this work, fibers so obtained will be referred to as long and short fibers, respectively. Dicumyl peroxide (DCP) at 98% was purchased from Sigma-Aldrich, Saint-Quentin Fallavier, France. Both acetone used for fiber impregnation with DCP and dichloromethane (CHCl_2_) used for the separation of the fibers from the matrix were provided by Carlo Erba Reagents srl, Italy, France. Chloroform (CHCl_3_) used for polymer extraction was purchased by VWR Chemicals, Fontenay-sous-Bois, France.

### 2.2. Chemical Treatment of Miscanthus giganteus Fibers

The surface of *Miscanthus giganteus* fibers was modified using the dicumyl peroxide (DCP). All kinds of fibers were dried at 80 °C in a conventional oven for 4 h, and then impregnated in a solution (8 mg/mL) of DCP in acetone. The quantity of DCP was varied from 0 to 0.25 wt % and 2.2 wt % of the total mass used during the compounding step. Solutions were stirred for 30 min at 200 rpm, and fibers were then dried statically before the realization of composites, until total evaporation of the solvent. This last step was evaluated by gravimetric analysis using a AG204 Mettler Toledo (Mettler Toledo, Viroflay, France) precision balance.

### 2.3. Composite Manufacturing

Before processing, PHBHV pellets and raw fibers were dried under vacuum at 80 °C for 4 h in order to avoid the presence of moisture during the mixing step. Composites with two different content of fibers (20 and 5 wt %) were realized by mixing together the matrix and the fibers, modified or not, in a lab-scale twin-screw extruder (Minilab Thermo Scientific Haake of Thermo Fischer Scientific, Illkirch, France). The experiments were performed at 160 °C (*T_E_*) with a screw speed of 60 rpm (n). The retention time for the pure matrix was 1 min; this time was increased to 2 min in order to fully disperse the fibers into the matrix. After recirculation, the molten material was shot in a microinjection unit (MiniJet Thermo Scientific Haake of Thermo Fischer Scientific, Illkirch, France) at a variable pressure, depending on the fiber weight fraction applied for 30 s. A maintenance pressure, lower than that used during the phase of injection, was applied for other 30 s. The collector and the mold temperatures were set at 165 °C (*T_I_*) and 45° (*T_m_*), respectively. The injection pressure was adjusted according to the increase of the polymer melt viscosity with the fiber content, in order to have entire specimens of 60 mm × 20 mm × 1 mm. 

### 2.4. Materials Characterization

#### 2.4.1. Gel Fraction

To derive a fine analysis of the mechanical behavior of the composite, it is necessary to quantify the gel (crosslinked matrix) fraction. That is why, after processing, the gel content for the composite PHBHV_80_MIS_20_ (DCP) was evaluated by solvent extraction. Samples (2 g) were extracted with 150 mL of CHCl_3_ using a Soxhlet for 24 h. The solvent extracted the non-reacted PHBHV, while gel and fibers were recovered after extraction, and dried until recovering constant weight. The knowledge of the total mass (gel + fibers) and the knowledge of the fibers mass fraction present in the composite, allow for calculating the exact weight of the gel, and as a consequence, its percentage by gravimetric analysis, using the following expression:

(1) $$Gel\;(\%)=\frac{W_{gel}}{W_{0}}\;100,$$

where *W_gel_* and *W*_0_ are, respectively, the dry weights of isolated gel and of initial material evaluated using an AG204 Mettler Toledo precision balance.

#### 2.4.2. Mechanical Testing

**Figure A1:**
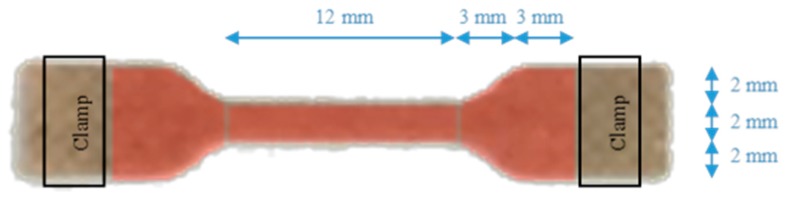
Geometry of the tested samples.

Tensile modulus, tensile strength, and failure strain for all realized composites were evaluated using an Instron 5965 Universal Testing Machine (Instron, Stockholm, Sweden) equipped with a load cell of 100 N. All “10 dumb-bell specimens” types were realized using a die cutter on the original specimens (with dimensions of 60 mm × 20 mm × 1 mm) obtained after injection molding processing. Specimens of standard dimensions according to ASTM D638 were then stored at 23 °C before testing 8 days after the realization day (see Figure A1 in the Appendix A). The mechanical characteristics of composites were evaluated by tensile tests carried out at a speed of 5 mm/min. At the end of the mechanical procedures, curves were averaged in order to obtain one averaged curve representative of each type of composite. 

#### 2.4.3. Scanning Electron Microscopy (SEM)

SEM observations were performed on the fracture sections of composites using a JEOL JSM6301F (JEOL, Croissy, France) scanning electron microscope. Prior to observation, the cross sections of analyzed specimens were sputter-coated with a thin layer of gold. Images were recorded with an acceleration voltage of 20 keV at a working distance of 15 mm.

#### 2.4.4. Fourier Transform Infrared Spectroscopy (FTIR)

Specimens of PHBHV/MIS and PHBHV/MIS/DCP composites realized with fibers of 1 mm length were solubilized in dichloromethane in order to separate fibers from matrix. Then, the collected fibers were extracted 3 times in 100 mL of dichloromethane at 54 °C, stirring for 30 min at 200 rpm. Fibers were then dried before analysis. Infrared spectra of the extracted fibers were recorded using a TENSOR27 Bruker (Bruker, Champs sur Marne, France) apparatus equipped with an attenuated internal reflection accessory using a diamond crystal (Digi Tech DLATGS Detector, (Bruker, Champs sur Marne, France, 32 scans, 4 cm^−1^) in the range 500–4000 cm^−1^. These spectra were then compared with that of raw *Miscanthus* fibers. In order to quantify the grafting of PHBHV on the MIS fibers due to the DCP, the ratio *R*_1_ was calculated as follows:

(2) $$R_{1}=\frac{I_{{1726\mathrm{cm}}^{-1}}}{I_{{1604\mathrm{cm}}^{-1}}},$$

where *I*_1726_ corresponds to the intensity of carbonyl group of PHBHV, and *I*_1604_ corresponds to the intensity of the esters present in the lignin structure. A measure of crystallinity was also evaluated using another index, the crystallinity index (*CI*) [50], which is the ratio of intensity of the band sensitive to crystallization to the band insensitive to the crystallization, in our case defined as follows:

(3) $$CI=\frac{I_{{1225\mathrm{cm}}^{-1}}}{I_{{1452\mathrm{cm}}^{-1}}},$$

where *I*_1225_ is assigned to the C–O–C stretching mode of the crystalline parts, and *I*_1452_ corresponds to the asymmetric deformation of the methylene groups. 

#### 2.4.5. Differential Scanning Calorimetry (DSC)

Differential scanning calorimetry experiments were performed on a PerkinElmer Diamond DSC Apparatus. Samples of around 10 mg sealed in aluminum pans were initially heated from −60 to 200 °C at 20 °C/min, cooled down rapidly, and then reheated in the same conditions used in the first heating run. Melting point (*T_M_*) and melting enthalpy (Δ*H_M_*) were determined during the first heating. The degree of crystallization (*X_c_*) was then calculated using the following equation:

(4) $$X_{c}(\%)=\frac{\Delta H_{M}}{\Delta H_{0}\times W}\times 100,$$

where Δ*H*_0_ corresponds to the melting enthalpy of 100% crystalline PHBHV (146 J/g) [62], and *W* is the PHBHV weight fraction present in each blend realized. 

#### 2.4.6. X-ray Diffraction (XRD)

Structural characterizations of *Miscanthus* fibers and PHBHV/MIS composites with and without DCP were determined by X-ray diffraction (XRD) using a D8 advance Bruker diffractometer (Bruker, Wissenbourg, France) operating at 40 kV and 40 mA with a CuKα radiation. The whole area investigated was in the range 2*θ* ≈ 5–40° at a scanning rate of 0.2°/min.

## 3. Results

### 3.1. Evaluation of PHBHV Grafting onto MIS Surface during Processing Evaluated by FTIR-ATR Analysis

During the extrusion process, the peroxide decomposes, creating free radicals that can react with the macromolecular chains of the PHBHV, forming ternary radicals. Free radicals can react with vegetal fiber constituents or combine with each other, with a crosslinking effect on the PHBHV. In this work, DCP was not directly added into the extruder but fibers were previously impregnated with DCP, and then processed in the extruder with the matrix. This procedure was preferred, in order to promote the grafting effect instead of crosslinking of the matrix. Using this technique, different biocomposites were prepared, varying the content of fibers (5 and 20 wt %), the length of fibers (1 mm and 45 µm), and the content of DCP (0, 0.25 and 2.2 wt %). All realized composites are listed in Table 1.

**Figure 1:**
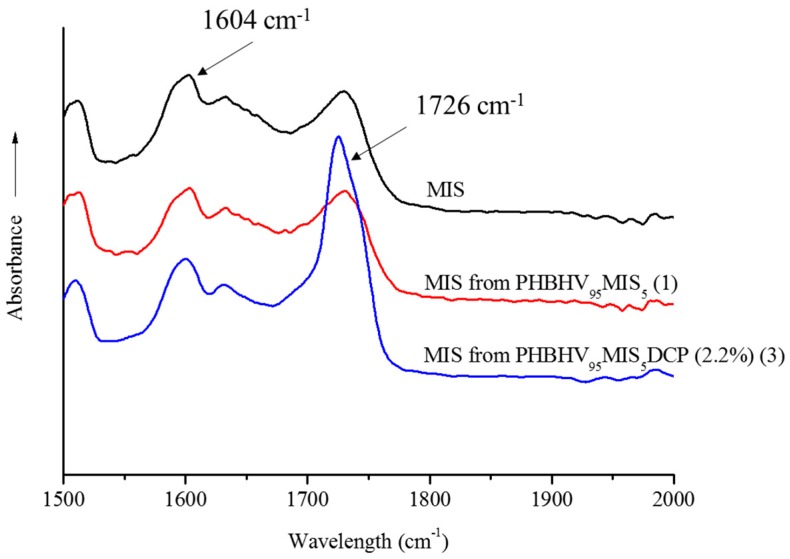
FTIR-ATR spectra of MIS, MIS extracted from the composite PHBHV_95_MIS_5_ (sample **1**), and MIS extracted from the composite PHBHV_95_MIS_5_ DCP (2.2 wt %) (sample **3**).

In order to show the presence of PHBHV chains grafted onto MIS surface, FTIR-ATR spectroscopy analyses were carried out on the fibers extracted from biocomposites PHBHV_95_MIS_5_ realized in the presence and in the absence of DCP (samples **1** and **3**). The ratio (*R*_1_) between the peak at 1726 cm^−1^, corresponding to the carbonyl group of PHBHV, and the peak at 1604 cm^−1^, corresponding to the ester groups of lignin, was evaluated. Figure 1 shows the superposition of FTIR-ATR spectra, the first of raw fibers, the second of fibers extracted from a composite with 5 wt % of fibers in the absence of DCP (sample **1**), and the last of fibers extracted from a composite with the same fiber charge and adding 2.2 wt % of DCP (sample **3**). 

The figure shows a significant peak at 1726 cm^−1^, typical of the carbonyl group of the matrix for the fibers that were treated in the presence of DCP. This qualitative result suggests that the grafting of PHBHV onto MIS surface occurred. As shown in Table 2, the ratio *R*_1_ increases with the increasing quantity of DCP. This fact means that more PHBHV was grafted onto the surface of the fibers. When DCP is not used, no PHBHV was grafted onto the MIS surface, and the ratio *R*_1_ is the same of that obtained for the raw *Miscanthus*.

**Figure 2:**
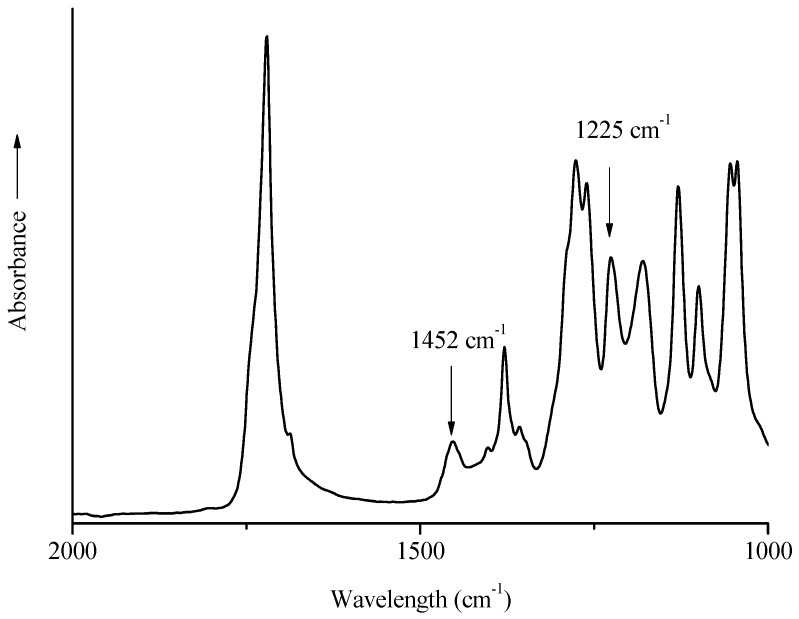
FTIR-ATR spectrum of a PHBHV specimen in the range 2000–1000 cm^−1^.

FTIR is also a useful analysis to evaluate the crystallinity of the PHBHV after processing. The band at 1726 cm^−1^ is representative of the C=O stretch present in the highly crystalline structure of the matrix, while the small shoulder at 1740 cm^−1^ represents the same stretch in the amorphous region. The band around 1378 cm^−1^ corresponds to the symmetrical wagging of the CH_3_ groups, and that at 1452 cm^−1^ to the asymmetric deformation of methylene groups. These bands are considered as insensitive to crystallinity, and they can be used to evaluate the crystallinity degree [16,50]. In particular, the bands at 1452 and 1225 cm^−1^, the latter corresponding to C–O–C stretching, were taken into account, to calculate the crystallinity index (see Figure 2). This index provides qualitative information about all the changes that may occur in the crystalline structure of the matrix. 

The CI decreased from 1.07 for the neat matrix to 1 for a composite with 20 wt % of raw fibers (Table 3). Compared to PHBHV/MIS blends, DCP treatment reduced the crystallinity index to 0.94 for a composite with 20 wt % of fibers. However, the decrease in the CI values caused by both fiber length and by the presence of DCP cannot be considered significant.

### 3.2. Tensile Properties

Biocomposites of different fiber content, length, and DCP percentage were realized by extrusion and injection molding, and the results of the mechanical tests are summarized in Table 4. The incorporation of 5 wt % of raw fibers causes an increase in tensile modulus from 889 MPa (value for the neat matrix) to 1074 MPa (sample **1**). This increase is more significant for the composites realized with 20 wt % of fibers, reaching the value of 1525 MPa when fibers of 45 µm were used (sample **9**). For biocomposites with 5 wt % of fibers realized in the presence of DCP, a decrease in tensile modulus is observed for a high content of DCP (samples **3** and **6**), compared to their equivalent realized with raw fibers (samples **1** and **4**). DCP is known to cause the crosslinking of the matrix that should exhibit an increase in tensile modulus [51]. Although the increase is not overly high, this effect is visible only for the composite PHBHV_80_MIS_20_ (sample **8**). A possible explanation for the decrease in tensile modulus might be the decrease in molar mass due to the presence of a high content of DCP. In order to verify this hypothesis, PHBHV was treated with DCP and then extruded and injected following the same procedure for processing of the neat matrix. The tensile modulus and the molar mass were then evaluated by tensile tests and size exclusion chromatography analysis. The PHBHV showed a decrease in tensile modulus from 889 to 782 MPa, and a decrease in molar mass from 100,000 to 54,000 g·mol^−1^, indicating that the degradation of the matrix occurred. In the case of the composite PHBHV_80_MIS_20_ (sample **8**), which exhibits an opposite trend, it may be possible that the high fiber content has a positive effect on the mechanical seal of the biocomposite with DCP, reducing the molecular chains’ scission, and improving the fibers/matrix interactions.

**Figure 3:**
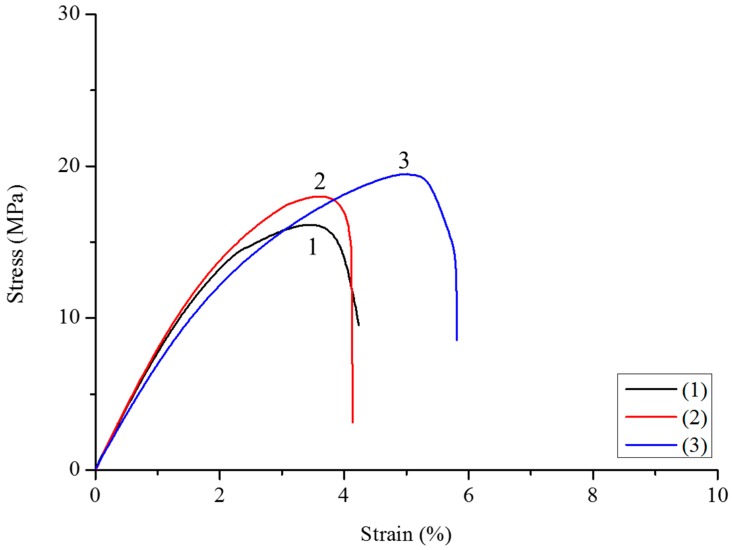
Strain–stress curves of composites PHBHV_95_MIS_5_ (sample **1**), PHBHV_95_MIS_5_ with 0.25 wt % of DCP (sample **2**), and PHBHV_95_MIS_5_ with 2.2 wt % of DCP (sample **3**).

**Figure 4:**
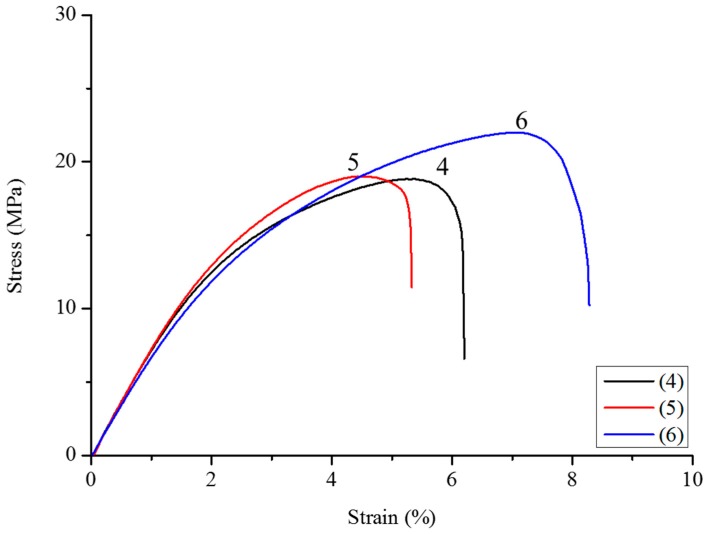
Strain–stress curves of composites PHBHV_95_MIS_5_ (sample **4**), PHBHV_95_MIS_5_ with 0.25 wt % of DCP (sample **5**), and PHBHV_95_MIS_5_ with 2.2 wt % of DCP (sample **6**).

**Figure 5:**
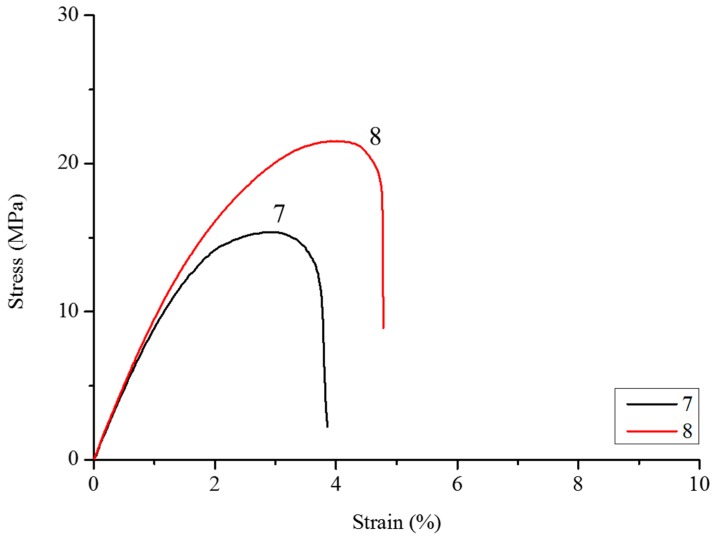
Strain–stress curves of composites PHBHV_80_MIS_20_ (sample **7**) and PHBHV_80_MIS_20_ with 2.2 wt % of DCP (sample **8**).

It is well known that the incorporation of vegetal fibers causes an increase in Young’s modulus, and at the same time, a decrease in final strength [24,63,64]. This fact is due to a weak interaction between the PHBHV and MIS fibers, impeding stress transfer in the two-phase interface. To improve the adhesion of the fibers to the matrix, *Miscanthus* fibers were modified with different amounts of DCP, as described previously, and the optimal DCP content was determined after the results obtained by traction tests. At a low content of DCP, typically 0.25 wt %, there is a slight increase in the final strength (samples **2** and **5**), and this result is independent from the length of the fibers used, as shown in Figure 3 and Figure 4. A content of DCP of 2.2 wt % is sufficient to improve the maximum strength and final strain for all composites, and in particular, for composites with 20 wt % of fibers, whose tensile strength passes from 15.8 (sample **7**) to 22 MPa (sample **8**), as shown also by Figure 5. When 5 wt % of DCP is used, the molten material cannot be extruded, because it undergoes an important crosslinking phenomenon, blocking the material in the recirculation zone of the extruder. A content of 2.2 wt % seems to be a good compromise between the grafting effect and the crosslinking phenomena.

We can affirm that the improvement of the stress transfer between the matrix and the reinforcement can be achieved by using fibers of 45 µm, but also by using DCP during the extrusion processing. Actually, the use of DCP allows for better mechanical properties for the biocomposites. However, at a high content of fibers, typically 20 wt %, the effect of fiber length is not visible on the final strength (sample **9**). Moreover, for the same composition, the presence of DCP prevents the realization of biocomposites, due to a strong interaction between fibers (sample **10**). The reason is that the total surface area exposed from these particles is very high, and in the presence of DCP, the molten material undergoes rapid fiber–fiber interactions, accompanied also by a crosslinking phenomenon, the latter preventing the realization of specimens by extrusion and injection molding in the same conditions as the other composites.

The composite PHBHV_80_MIS_20_ containing 2.2 wt % of DCP and 20 wt % of long fibers (sample **8**) was judged as the optimum composition. In this context, the degradation of the neat matrix, in the presence of DCP, was strongly limited by the great number of fibers. All the mechanical results are listed in the Table 4. The Soxhlet extraction on this composite has led to the determination of a crosslinked portion of 23 wt % on the total matrix present in the specimens. Assuming a quasi-constant density of the matrix if crosslinked or not (1.3 g/cm^3^), this fraction was determinant in the following paragraph to determine the effective mechanical properties of this composite.

### 3.3. Fracture Facies Morphology

**Figure 6:**
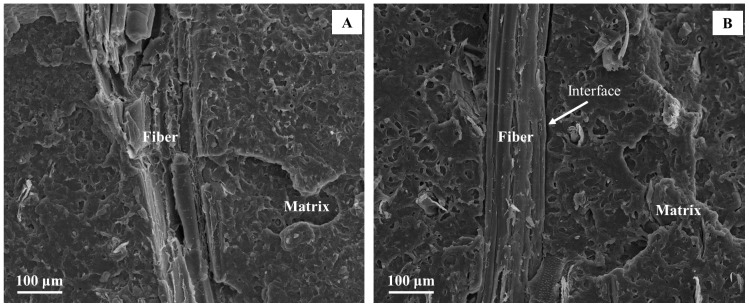

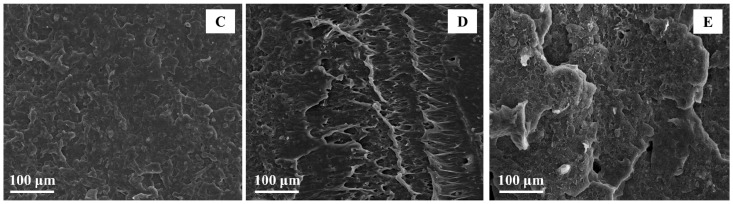
SEM images of PHBHV_95_MIS_5_ composites realized with fibers of 1 mm treated with DCP (**A**) and untreated (**B**) pure PHBHV (**C**) and of the matrix in the PHBHV_95_MIS_5_ (DCP) composites realized with fibers of 1 mm (**D**) (sample **3**) and 45 µm (**E**) (sample **6**).

After traction tests, the fracture section of each specimen was observed by SEM in order to evaluate the adhesion between the fibers and the matrix. These observations revealed two different effects due to the presence of the DCP as deduced from mechanical tests. The first one is the improvement of the adhesion between matrix and fibers, shown in Figure 6A. Indeed, when considering untreated fibers (cf. Figure 6B), a non-cohesive interface can be observed. This indicates that the increase in ultimate strength for treated fibers is due to an improved stress transmission between the composite different phases during the traction test. 

The second effect is less evident, and a zoom on a specific part of the specimen where only matrix is present evaluated it. In these zones, there is an evident structural change of the pure matrix. This morphological change could be due to a crosslinking effect caused by the presence of DCP. These zones are not homogeneous in all sections. Moreover, the type of crosslinking is different if long fibers are used instead of short fibers. In the case of composites realized with short fibers, the resulting network seems to be more compact than that obtained in the presence of long fibers (Figure 6D,E). This fact supports the mechanical results in which composites with 5 wt % of short fibers present better mechanical properties than their equivalent with long fibers and composites with 20 wt % of short fibers cannot be extruded. 

### 3.4. Characterization of Biocomposites by DSC and XRD Analyses

**Figure 7:**
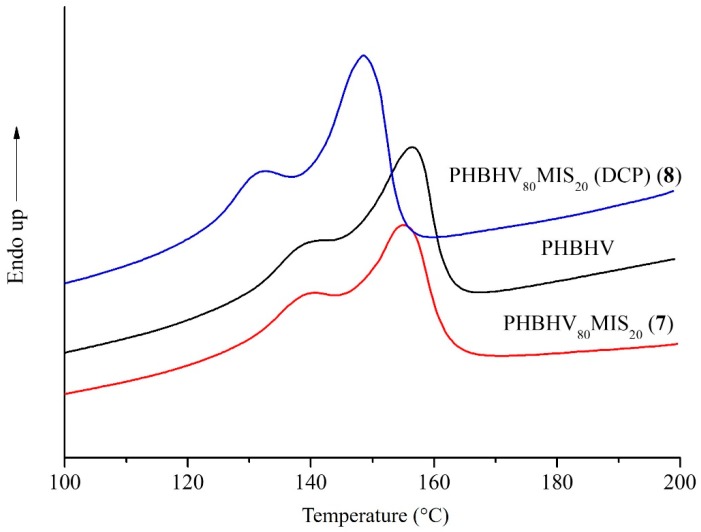
DSC first heating thermograms of PHBHV, PHBHV_80_MIS_20_ (sample **7**), and PHBHV_80_MIS_20_ (DCP) (sample **8**).

The differential scanning calorimetry (DSC) was used in order to evaluate the thermal behavior of the final composites. All the curves of the composites present two melt peaks due to the repartition of crystallites of different dimensions typical of semi-crystalline polymers (Table 5). Two different melt temperatures (T_M1_ and T_M2_) characterize each peak. Comparing the neat matrix and composites realized with high content of raw fibers, typically 20 wt % (sample **7**), no modification of thermal properties was detected. This fact means that *Miscanthus* fibers do not have an impact on the crystallization behavior of the matrix. The presence of DCP during the processing step causes a modification in melt temperatures, the latter passing from 156 to 148 °C (T_M2_), and from 140 to 133 °C (T_M1_) for a composite realized with 20 wt % of fibers in the presence of 2.2 wt % of DCP (sample **8**) as shown in Figure 7. At the same time, no change in crystallinity was detected for the same sample. This result could be explained by the fact that DCP does not alter the semicrystalline behavior of the matrix, whose crystallinity degree remains at 31%, but it could have a significant impact on the crystallite’s size and shape.

**Figure 8:**
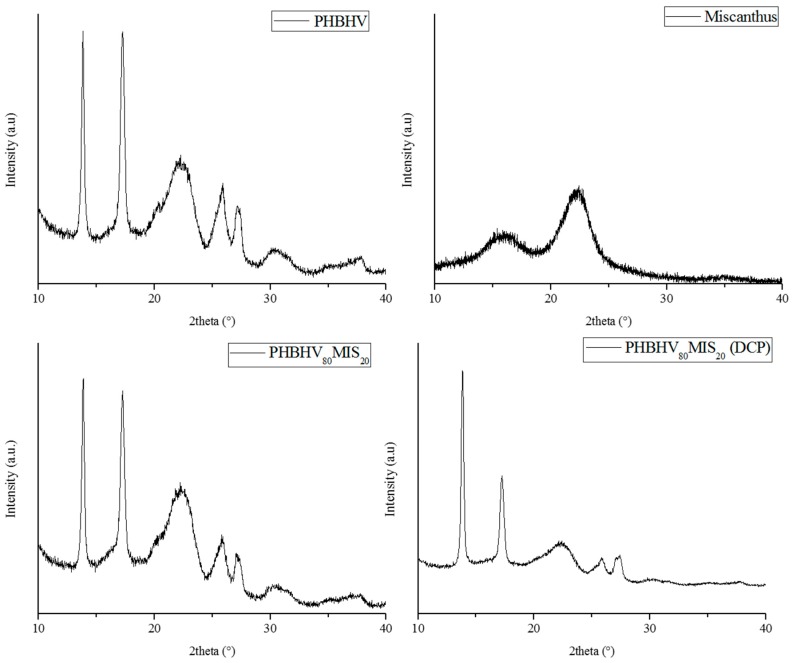
XRD diffractograms of MIS, PHBHV, PHBHV_80_MIS_20_ (sample **7**), and PHBHV_80_MIS_20_ (DCP) (sample **8**).

This shift at lower fusion temperature suggested a change in the crystallite’s dimensions. XRD analysis was also conducted on the neat matrix and composites with 20 wt % of long fibers realized with and without DCP (Figure 8). PHBHV has a semicrystalline nature, with characteristic peaks at 2θ around 13°, 17°, 21°, 22°, 25° and 27°, corresponding to planes (020), (110), (101), (111), (121) and (040), respectively, in the orthorhombic crystalline lattice. The addition of MIS and DCP does not alter the basic crystal structure of PHBHV, being the reflections located at the same angle. Moreover, the evaluation of Bravais parameters showed that the lattice volume did not change.

### 3.5. Analytical Models

Our goal is now to provide, in this section, modeling approaches that, if they can mimic the actual behavior of the composites, could then be used to provide an in silico design of composites according to various applications. To this aim, we used micromechanics analytical approaches [65,66,67,68]. In these models, a perfect contact between the inclusions and the matrix was assumed. According to Figure 6, this assumption seems relevant. 

#### 3.5.1. Use of a Model Involving Three Phases

The rule of mixtures (ROM), and the Mori–Tanaka model (MT), are ways to analytically derive the effective Young’s modulus of a multiphasic medium. Concerning the latter one, it is based on Eshelby’s elasticity solution for diluted particle inclusion in an infinite matrix [68]. These two methods require one to properly describe the different components that form the composite (indexed “*C*”). Here, we chose to consider three phases: the fibers (indexed “*F*”), the gel (crosslinked matrix, indexed “*G*”), and the remaining matrix (indexed “*M*”). To apply these methods, it is necessary to know the volume fraction *Φ_i_*, and the bulk Young moduli *E_i_* of each phase (*i* = *F*, *G* or *M*). Thus, the Young’s modulus of the composite *E_c_* can be described through two functions of these 6 parameters:

(5) $$E_{\mathrm{ROM}}=\sum^{\text{​}}\Phi_{i}E_{i}=f_{\mathrm{ROM}}{(E}_{F}{,\;E}_{G}{,\;E}_{M}{,\;\Phi }_{F}{,\;\Phi }_{G}{,\;\Phi }_{M}),$$

(6) $$E_{\mathrm{MT}}=f_{\mathrm{MT}}{(E}_{F}{,\;E}_{G}{,\;E}_{M}{,\;\Phi }_{F}{,\;\Phi }_{G}{,\;\Phi }_{M}),$$

which respectively correspond to the mixing rule or the Mori–Tanaka approach. Knowing five of these six parameters and the effective Young’s modulus of the composite, it is thus possible to recover the sixth parameter. Note that the conservation of the volume may reduce the number of unknown volume fractions, since their sum is one. We decided to focus our attention on PHBHV_80_MIS_20_ (DCP) (sample **8**), and apply to this peculiar specimen, the two methods (Mori–Tanaka and rule of mixtures) to evaluate its elastic modulus. To this aim, the fraction of gel *Φ_G_* was calculated using the experimental procedure previously described (See Section 2.4.1). The value of *Φ_F_* can be easily deduced from fiber mass content and density. By this way, the knowledge of two volume fractions provides the last one thanks to the volume conservation law (*Φ_F_* + *Φ_G_* + *Φ_M_* = 1). The Young’s modulus of the crosslinked matrix was evaluated experimentally, by testing specimens of neat matrix realized in the presence of DCP (See Section 3.2). The final values used to implement the models are resumed in Table 6.

#### 3.5.2. Evaluation of *E_G_* and *Φ_G_* by a Mathematical Approach

The application of the rule of mixtures to composites, realized in the absence of DCP, showed discrepancies between the real value of Young’s modulus and that obtained by ROM. In particular, for the composite PHBHV_80_MIS_20_ (sample **7**), the error between the experimental value and that obtained with the ROM is quite substantial, around 33%. This great difference could be due to the lack of knowledge of the fiber’s Young’s modulus, assumed to be equal to 4.5 GPa, this last value being taken from literature data. Moreover, possible stress concentration phenomena within the matrix may occur, and thus explain these discrepancies. 

The Young’s modulus of the crosslinked matrix is one of the principal unknowns of our system. At first, we apply an experimental procedure to calculate this value. If we consider that the matrix totally crosslinks during the manufacturing step, this hypothesis allows us to assimilate the Young’s modulus calculated for specimens of neat PHBHV, realized with DCP, to that of any crosslinked portion present in our composite. Then, assuming that the nature of the crosslinked portion does not change when fibers are present in the blend, it is possible to perform analytical and numerical approaches to estimate the effective modulus of the composite. However, the uncertainties related to the values of *E_F_*, *E_G_*, and to the fraction of crosslinked matrix cause the re-opening of the discussion about the value of moduli used in the models, and questions about the role of vegetal fibers in the blend when DCP is present. 

For this reason, at this step, we decided to use MATLAB R2007b software to map the variation of *E_G_* as a function of the crosslinked fraction *Φ_G_*, by implementing a function F from the function f_ROM_ defined by Equation (5). If the values of *E_G_* and *Φ_G_* are variable (*E_G_* varied from 0 to 2 GPa and *Φ_G_* from 0 to 1 − *Φ_F_*), the other parameters are those obtained through experimental procedures. The function *F* reads as follows:

(7) $${F(\Phi }_{G(j)}{,E}_{G(i)}{)=\Phi }_{F}E_{F}{+\Phi }_{G(j)}E_{G(i)}+(1-\Phi_{F}-\Phi_{G(j)}{)E}_{M}-E_{\exp}.$$

Assuming the accuracy of the mixing rule, the zero-curve obtained from this function provides possible couples ($\Phi_{G}{,\;E}_{G})$ checking the experimental effective behavior. Note that this value is characterized by errors, due to the standard deviation associated with *E_exp_*, and to the assumptions made about *E_F_*.

#### 3.5.3. Results of Modeling Approaches

The results of analytical methods on the composite PHBHV_80_MIS_20_ (DCP) (sample **8**) are presented in Table 7. The homogenized models with cylindrical and spherical inclusions constituted the upper and lower boundary for the experimental values, the first one representing a fully oriented configuration, whereas the second one remaining isotropic.

According to the results in Table 6, models provide unrealistic values compared to the experimental ones. This fact means that the uncertainties presented in the previous paragraph concerning the Young’s modulus of the crosslinked matrix and the choice made on that of vegetal fibers have a great impact on the determination of the elastic modulus of a partially crosslinked composite. Moreover, these discrepancies between the experimental and approximated moduli can be, in part, explained by the bad repartition of the fibers within the matrix volume [70].

**Figure 9:**
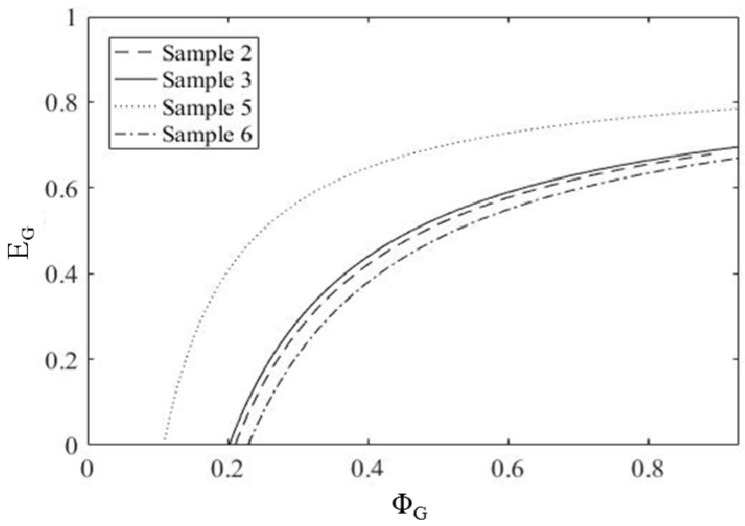
Evaluation of *E_G_* as function of *Φ_G_* for composites PHBHV_95_MIS_5_ (samples **2**, **3**, **5** and **6**).

The mapping operation conducted on the specimens of PHBHV_95_MIS_5_ (samples **2**, **3**, **5** and **6**) presents the various lines of possibility. When considering fully crosslinked matrix, that is to say, asymptotic values which correspond to the total crosslink of the matrix, it remains more or less the same, notwithstanding the DCP concentration (See Figure 9). Moreover, the Young’s modulus *E_G_* found for these composites ranges between 0.6 and 0.8 GPa. These values agree with the experimental modulus (0.782 GPa) previously evaluated on specimens of neat matrix totally crosslinked. More precisely, lower values of modulus are obtained for higher DCP content (samples **3** and **6** were realized with 2.2 wt % of DCP), while higher values of modulus for lower DCP content (samples **2** and **5** were realized with 0.25 wt % of DCP).

This result indicates that the crosslinking phenomenon within the matrix is predominant when compared to the grafting of fibers. 

**Figure A2:**
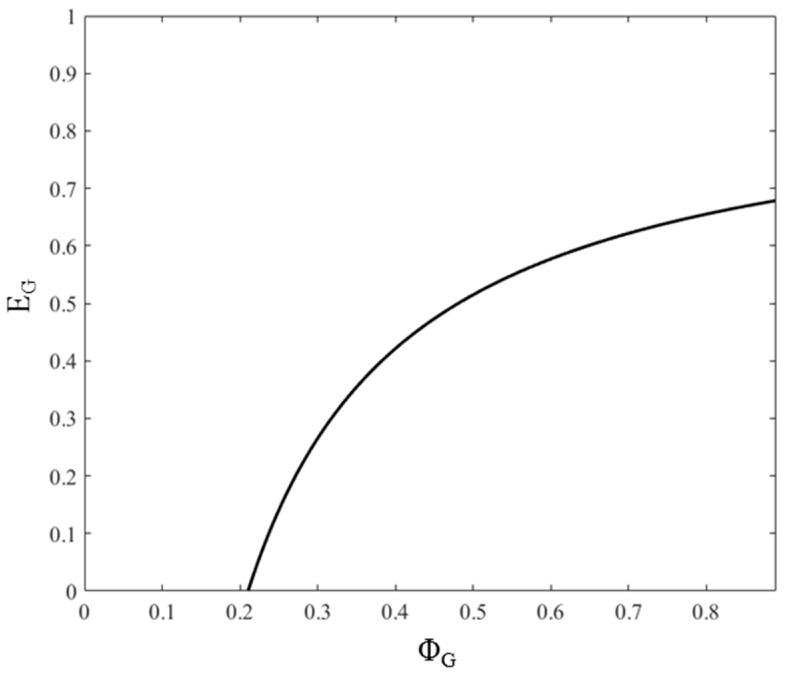
Evaluation of *E_G_* as a function of *Φ_G_* for composite PHBHV_90_MIS_10_ (DCP).

For all described composites, it seems that the mechanical behavior oscillates from one (typical of specimens with 5 wt % of fibers) in which the content of fibers is too low to have a remarkable impact on the stiffness of the material, to another (typical of specimens with 20 wt % of fibers) in which the presence of high content of reinforcement influences not only the mechanical behavior, but also the reaction of DCP. This threshold effect, due to the content of fibers, was verified *a posteriori*, realizing PHBHV-based composites with 10 wt % of short MIS fibers and 2.2 wt % of DCP (see Appendix for more details, Table A1, Figure A2). The properties obtained for the additional specimen demonstrate that the composite is similar to those realized with 5 wt % of fibers in terms of mechanical behavior and in terms of crosslinking phenomena. Moreover, the fact that the realization of this composite by extrusion and injection molding was possible is a further clue that the high fiber content behavior was not reached for this composite (the processing at 20 wt % of short fibers being unrealizable). 

## 4. Conclusions

Free radical grafting initiated by DCP via reactive extrusion led to the improvement of the mechanical performances of PHBHV/MIS composites. A total amount of 2.2 wt % of DCP was found to be the optimum content, in order to achieve a good adhesion of fibers/matrix with consequent improvement in final strength and elongation at the break of the biocomposites. Fibers sieved with a 45 μm mesh sieve showed, in general, a better improvement of the mechanical properties. Although this is a good result, the great interfacial area exposed by these little particles caused a limitation in the processing step when a lot of these fibers were used. This fact led to the choice of the blend with 20 wt % of long fibers and 2.2 wt % of DCP as the better solution, realized in terms of the mechanical results obtained, less matrix degradation during the process, and no significant change in crystallinity degree, due to the presence of fibers or DCP. The evidence of the grafting between the fibers and the matrix was proven by FTIR-ATR and SEM analyses, and the knowledge of the gel fraction for this composite led to the implementation of analytical models. However, the Mori–Tanaka model with 3 phases (neat matrix/crosslinked matrix/fibers) overestimates the mechanical behavior of the composite. The mathematical approach used to evaluate the ranges of Young’s modulus of the crosslinked matrix and of its volumetric fraction agree with the experimental values. This result is not true for higher fiber contents (typically 20 wt %), in which the presence of a lot of fibers influences the normal reaction of DCP. A threshold effect (between 10 wt % and 20 wt %), correlated to the content of fibers, is visible on the analyzed specimens. The first behavior is that typical of a material without fibers, while the second one is of a composite with a high content of fibers that is able to modify the normal reaction occurring between DCP and PHBHV. The possibility to substitute a part of the matrix with vegetal fibers, the improvement of the adhesion of fiber/matrix, and the possibility to process these composites with conventional techniques, suggest that the functionalization of natural fibers with DCP appears to be a very promising way to improve the mechanical properties of any type of polyesters.

## Figures and Tables

**Table 1:** Composition of PHBHV/MIS (poly(3-hydroxybutyrate-*co*-3-hydroxyvalerate/*Miscanthus giganteus* fiber) composites at different fiber contents (5 and 20 wt %), length (1 mm and 45 µm), and dicumyl peroxide (DCP) content (0, 0.25 and 2.2 wt %).

Sample	PHBHV_x_MIS_1−x_	MIS Length	DCP (wt %)
	PHBHV_100_	-	0
1	PHBHV_95_MIS_5_	1 mm	0
2	PHBHV_95_MIS_5_	1 mm	0.25
3	PHBHV_95_MIS_5_	1 mm	2.20
4	PHBHV_95_MIS_5_	45 µm	0
5	PHBHV_95_MIS_5_	45 µm	0.25
6	PHBHV_95_MIS_5_	45 µm	2.20
7	PHBHV_80_MIS_20_	1 mm	0
8	PHBHV_80_MIS_20_	1 mm	2.20
9	PHBHV_80_MIS_20_	45 µm	0
10	PHBHV_80_MIS_20_	45 µm	2.20

**Table 2:** Comparison of *R*_1_ values obtained by FTIR-ATR analysis as function of DCP content for fibers of 1 mm raw and extracted from biocomposite PHBHV_95_MIS_5_ (samples **1** and **3**).

Sample	PHBHV*_x_*MIS_1−*x*_	DCP (wt %)	$R_{1}=\frac{I_{1726{\mathrm{cm}}^{-1}}}{I_{1604{\mathrm{cm}}^{-1}}}$
	MIS_100_	--	1.3 ± 0.01
1	PHBHV_95_MIS_5_	0	1.3 ± 0.01
2	PHBHV_95_MIS_5_	0.25	1.6 ± 0.02
3	PHBHV_95_MIS_5_	2.20	3.4 ± 0.04

**Table 3:** Crystallinity parameters of PHBHV and its composites determined by FTIR-ATR analysis.

Sample	PHBHV_x_MIS_1-x_	MIS Length	DCP (wt %)	CI
	PHBHV_100_		0	1.07 ± 0.1
1	PHBHV_95_MIS_5_	1 mm	0	1.06 ± 0.2
3	PHBHV_95_MIS_5_	1 mm	2.2	0.98 ± 0.1
4	PHBHV_95_MIS_5_	45 μm	0	1.00 ± 0.1
6	PHBHV_95_MIS_5_	45 μm	2.2	0.95 ± 0.3
7	PHBHV_80_MIS_20_	1 mm	0	1.00 ± 0.1
8	PHBHV_80_MIS_20_	1 mm	2.2	0.94 ± 0.2

**Table 4:** Tensile properties of biocomposites determined by tensile tests.

Sample	PHBHV*_x_*MIS_1−*x*_	MIS Length	DCP (wt %)	*E* (MPa)	*σ_max_* (MPa)	*ε_r_* (%)
	PHBHV_100_	-	-	889 ± 41	23.0 ± 0.5	11 ± 1.60
1	PHBHV_95_MIS_5_	1 mm	-	1074 ± 44	17.0 ± 1.7	4.5 ± 0.89
2	PHBHV_95_MIS_5_	1 mm	0.25	1126 ± 37	18.0 ± 0.7	4.1 ± 0.43
3	PHBHV_95_MIS_5_	1 mm	2.2	962 ± 19	20.0 ± 0.9	5.9 ± 0.74
4	PHBHV_95_MIS_5_	45 μm	-	1021 ± 20	18.0 ± 0.1	6.3 ± 0.27
5	PHBHV_95_MIS_5_	45 μm	0.25	1045 ± 62	20.0 ± 1.0	5.3 ± 0.60
6	PHBHV_95_MIS_5_	45 μm	2.2	938 ± 55	22.1 ± 0.4	8.3 ± 1.09
7	PHBHV_80_MIS_20_	1 mm	-	1267 ± 90	15.8 ± 0.7	3.9 ± 0.22
8	PHBHV_80_MIS_20_	1 mm	2.2	1358 ± 52	22.0 ± 1.0	4.8 ± 0.46
9	PHBHV_80_MIS_20_	45 μm	-	1525 ± 84	17.0 ± 0.7	3.1 ± 0.30
10	PHBHV_80_MIS_20_	45 μm	2.2	-	-	-

**Table 5:** DSC data for PHBHV/MIS composites realized with fibers of 1 mm and 45 μm: T_M1_ and T_M2_ (melt temperatures); Δ*H_M_* (melt enthalpy); *X_c_* (crystallinity degree).

Sample	PHBHV*_x_*MIS_1−*x*_	DCP (wt %)	MIS Length	T_M1_ (°C)	T_M2_ (°C)	Δ*H_M_* (J/g)	*X_c_* (%)
	PHBHV	-	-	140	156	46	31
1	PHBHV_95_MIS_5_	-	1 mm	140	154	45	32
3	PHBHV_95_MIS_5_	2.2	1 mm	137	154	44	32
4	PHBHV_95_MIS_5_	-	45 μm	140	159	48	35
6	PHBHV_95_MIS_5_	2.2	45 μm	138	156	43	31
7	PHBHV_80_MIS_20_	-	1 mm	141	155	37	31
8	PHBHV_80_MIS_20_	2.2	1 mm	133	148	40	31

**Table 6:** Technical data for Young’s modulus and volumetric fraction for *Miscanthus giganteus*, PHBHV, and gel fraction.

Constituents	Young‘s Modulus (Gpa)	Volumetric Fraction (%)
*Miscanthus giganteus*	4.5 ^(a)^	0.22
PHBHV	0.889 ^(b)^	0.60
Gel	0.782 ^(b)^	0.18 ^(c)^

^(a)^ Adapted from [69]; ^(b)^ experimental value; ^(c)^ evaluated according to procedure described in Section 2.4.1.

**Table 7:** Comparison between Mori–Tanaka model, ROM, and FE model, and the experimental value for the composite PHBHV_80_MIS_20_ (DCP) (sample **8**).

Sample	MT Model 3 Phases		ROM	Experimental
	*E_MT_cyl_* (Mpa)	*EMT_MT_sph_* (Mpa)	*E_ROM_* (Mpa)	*E_exp_* (Mpa)
**8**	1655	1170	1655	1358 ± 52

## Appendix A

### Appendix A1. ASTM D638 Standard Test Method for Plastics Tensile Properties

The geometry of the sample is designed according to the ASTM D638 standard test method for the tensile properties of plastics.

### Appendix A2. Realization of Specimens of PHBHV_90_MIS_10_ (2.2% DCP)

In order to demonstrate the threshold effect due to the fibers when the reaction of PHBHV with DCP occurred, we decide to realize a composite with an intermediate content of short fibers (between 5 wt % and 20 wt %) and 2.2 wt % of DCP. Note that the realization of a composite with 20 wt % of fibers and the same content of DCP was impossible, due to the excessive crosslinking effect in the extruder. Once realized, specimens were tested in the same conditions as the others (see Section 2.4.2 for details). Mechanical properties and results obtained by the mathematical approach are resumed in Table A1, and visible in Figure A2, respectively.

**Table A1:** Tensile properties of biocomposite PHBHV_90_MIS_10_ (DCP) determined by tensile tests.

Sample	*E* (Mpa)	*σ_max_* (Mpa)	*ε_r_* (%)
PHBHV_90_MIS_10_ (DCP)	1099 ± 65	22 ± 2.0	7 ± 1.0

The properties of PHBHV_90_MIS_10_ (DCP) composite are similar to those of composite realized with 5 wt % of fibers, in terms of mechanical behavior and in terms of crosslinking phenomena.
